# Closed-Form Algorithm for 3-D Near-Field OFDM Signal Localization under Uniform Circular Array

**DOI:** 10.3390/s18010226

**Published:** 2018-01-14

**Authors:** Xiaolong Su, Zhen Liu, Xin Chen, Xizhang Wei

**Affiliations:** College of Electronic Science, National University of Defense Technology, Changsha 410073, China; suxiaolong_nudt@163.com (X.S.); chenxin10@nudt.edu.cn (X.C.); liweier@nudt.edu.cn (X.W.)

**Keywords:** near-field OFDM signal, uniform circular array (UCA), sparse representation (SR), phase difference, parameter estimation

## Abstract

Due to its widespread application in communications, radar, etc., the orthogonal frequency division multiplexing (OFDM) signal has become increasingly urgent in the field of localization. Under uniform circular array (UCA) and near-field conditions, this paper presents a closed-form algorithm based on phase difference for estimating the three-dimensional (3-D) location (azimuth angle, elevation angle, and range) of the OFDM signal. In the algorithm, considering that it is difficult to distinguish the frequency of the OFDM signal’s subcarriers and the phase-based method is always affected by errors of the frequency estimation, this paper employs sparse representation (SR) to obtain the super-resolution frequencies and the corresponding phases of subcarriers. Further, as the phase differences of the adjacent sensors including azimuth angle, elevation angle and range parameters can be expressed as indefinite equations, the near-field OFDM signal’s 3-D location is obtained by employing the least square method, where the phase differences are based on the average of the estimated subcarriers. Finally, the performance of the proposed algorithm is demonstrated by several simulations.

## 1. Introduction

The orthogonal frequency division multiplexing (OFDM) signal has superior performance due to the practical applications in wireless local area networks (WLAN), 4G mobile communications, digital audio broadcasting (DAB) systems, radar, etc., which have received considerable attention in the field of source localization [1,2,3]. Uniform circular array (UCA) is an attractive geometry and is preferable over uniform linear array (ULA) because of its 360° azimuth coverage, additional elevation angle information and almost identical beamwidth in the context of three-dimensional (3-D) parameter estimation [4,5,6,7,8,9,10].

For the problem of near-field source localization, an improved 3-D Multiple Single Classification (MUSIC) method [7] was proposed to estimate the 3-D location. While the 3-D MUSIC method for a joint estimation of the azimuth, elevation angle, and range can cope with multiple sources, it requires an expensive 3-D search procedure. Based on the phase of the adjacent sensors’ correlation function, Jung et al. [8] presented a closed-form estimator for the 2-D direction of arrivals (DOAs) and range of a single narrowband source, which is more computationally efficient. In [9], by using a fixed rotary UCA with a center sensor, Chen et al. considered the condition of multiple mono-frequency sources and the ambiguity in the phase-based algorithm, the phase difference before and after rotation is utilized to obtain the 3-D localization and resolve ambiguity. However, it is limited by the specific condition that the frequency spacing should be more than 8 times frequency resolution, which cannot be extended to the localization for the wideband signal.

The aforementioned method has not been focused on the wideband source such as the OFDM signal localization in the near-field condition. Due to the fact that the OFDM signal has several subcarriers and the frequency of the adjacent subcarriers is closed to each other, it is difficult to distinguish the subcarriers in the frequency domain. Notwithstanding, padding zeroes at the tail of the discrete measurements is equal to interpolating in the frequency domain when the Fast Fourier Transformation (FFT) algorithm is performed, which can improve frequency resolution and avoid the phenomenon of spectrum leakage. Yet, it will lead to the deviation of frequency estimation and the inaccuracy of the corresponding phase difference [11]. By turning to the sparse representation (SR) framework, we are able to achieve super-resolution without a large number of time samples, and with lower sensitivity to signal-to-noise ratio (SNR) [12,13,14]. Therefore, the goal of this paper is to explore how to utilize the sparse representation methodology for the subcarriers’ frequency estimation.

Accordingly, under a fixed UCA, this paper presents a closed-form algorithm that extends the scheme in [8] to estimate the 3-D position of the OFDM signal. Herein, considering that the phase-based algorithm seriously suffers from the errors of the frequency and the corresponding phase estimation, we first employ sparse representation (SR) to decouple the subcarriers in the frequency domain and obtain the corresponding super-resolution phases. Moreover, we calculate the phase difference of the adjacent sensors for each subcarrier. Furthermore, as the phase differences including azimuth angle, elevation angle and range parameters can be expressed as indefinite equations, we make full use of subcarriers’ phase differences and employ the normalized phase differences of the subcarriers. Meanwhile, the least square method is utilized to obtain the near-field OFDM signal’s 3-D location. Simulation results are performed to illustrate the effectiveness of the proposed algorithm, which can estimate the super-resolution frequencies of the OFDM signal’s subcarriers and obtain the 3-D location of a near-field OFDM signal accurately.

## 2. Signal Model

**Figure 1:**
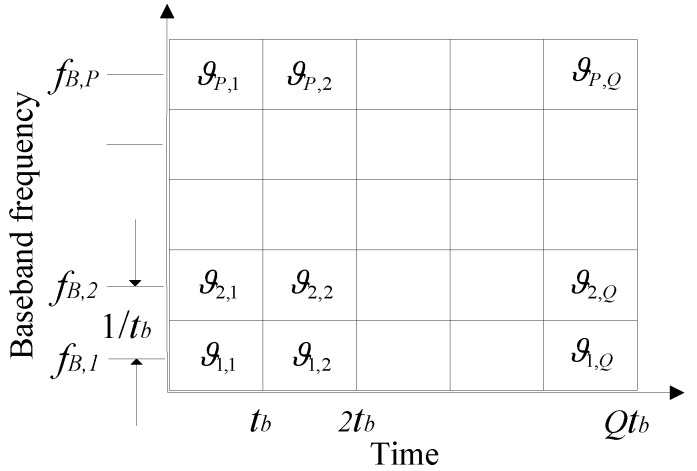
The structure of an OFDM signal in time domain.

After serial in parallel out (SIPO), the sequences become parallel streams, which are simultaneously modulated by different subcarriers with the same frequency interval. In order to guarantee that the subcarriers of the OFDM signal are orthogonal to each other, the frequency spacing of the adjacent subcarriers is set to the reciprocal of a code time width. The structure of the OFDM signal in time domain is shown in Figure 1.

For p=1,…,P, q=1,…,Q, where $\vartheta_{p,q}$ is the pth subcarrier’s phase within the duration of the qth elementary symbol, $t_{b}$ is the duration of the completed OFDM elementary symbol, $f_{B,p}$ is the baseband frequency of the pth subcarrier, and Δf is the frequency spacing of the adjacent subcarriers which is equal to the reciprocal of $t_{b}$.

Focusing on the frequency and phase, as the baseband frequency $f_{B,p}$ is processed by the up converter with the carrier frequency $f_{c}$, the OFDM signal within the duration of elementary symbol can be simplified as

(1) $$s(t)=\sum_{p=1}^{P}\left\{\exp(j2\pi f_{p}t)+\exp(j\vartheta_{p})\right\}$$

where $\left(q-1\right)t_{b}<t\leq qt_{b}$, $f_{p}$ represents the frequency of the pth subcarrier, and $\vartheta_{p}$ is the pth subcarrier’s phase.

**Figure 2:**
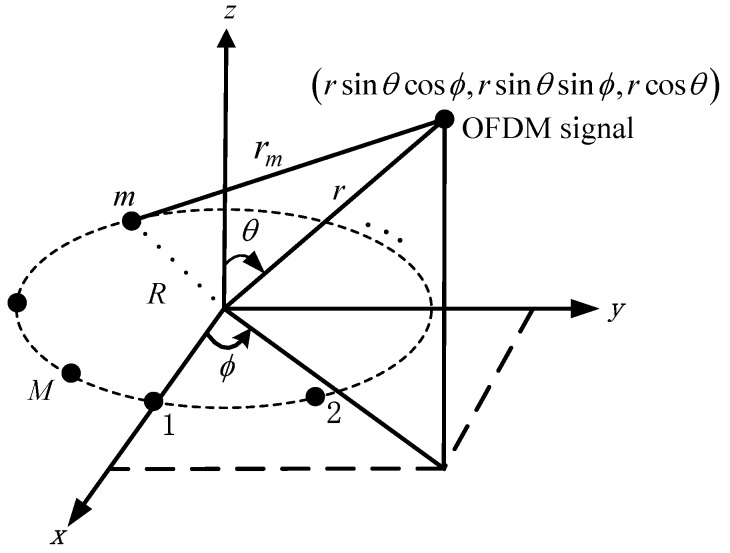
Geometry of a UCA with a near-field OFDM signal.

Consider a UCA in the xy-plane with radius R and M identical omnidirectional sensors impinged by an OFDM signal. The sensors are uniformly and counterclockwise spaced on the circumference where the first sensor is located at the *x*-axis, its geometry is shown in Figure 2. The near-field OFDM signal is located at (ϕ,θ,r), where the azimuth angle ϕ∈[−π,π) is measured counterclockwise from the *x*-axis, the elevation angle θ∈[0,π/2) is measured downward from the *z*-axis, and the range r is measured from the center of the UCA. The output of the mth sensor of the UCA at the nth sample is given by

(2) $$x_{m}^{}\left[n\right]=s\left[n\right]e^{\left(j2\pi /\lambda \right)\left\{r-r_{m}^{}(\phi ,\theta ,r)\right\}}+w_{m}^{}\left[n\right]$$

for m=1,2,…,M and n=1,2,…,N, where s[n] represents the OFDM signal sampling with power $\sigma_{s}^{2}$, and $w_{m}\left[n\right]$ is assumed to be a zero-mean white complex Gaussian noise with power $\sigma_{n}^{2}$, which is independent of s(n). λ is the wavelength. $r_{m}^{}(\phi ,\theta ,r)$ is the range between the mth sensor and the near-field OFDM signal, which has the form of

(3) $$r_{m}^{}(\phi ,\theta ,r)=\sqrt{r^{2}+R^{2}-2rR\zeta_{m}^{}(\phi ,\theta )}$$

where $\zeta_{m}^{}(\phi ,\theta )=\cos(\gamma_{m}^{}-\phi )\sin\theta$ with $\gamma_{m}^{}=2\pi (m-1)/M$. According to a second-order Taylor series expansion around the point where the value of $R/r_{p}$ is approximated to zero, $r_{m}(\phi ,\theta ,r)$ for m=1,…,M can be well simplified as

(4) $$r_{m}^{}(\phi ,\theta ,r)\approx r-R\zeta_{m}^{}(\phi ,\theta )+\left(R^{2}/2r\right)\left(1-\zeta_{m}^{2}(\phi ,\theta )\right)$$

Substituting (3) and (4) into (2) yields the approximated signal model

(5) $$x_{m}^{}\left[n\right]=s\left[n\right]e^{\left(j2\pi R/\lambda \right)\left\{\zeta_{m}^{}(\phi ,\theta )-\left(R/2r\right)\left(1-\zeta_{m}^{2}(\phi ,\theta )\right)\right\}}+w_{m}^{}\left[n\right]$$

## 3. Frequency Estimation of the OFDM Signal’s Subcarriers

**Figure 3:**
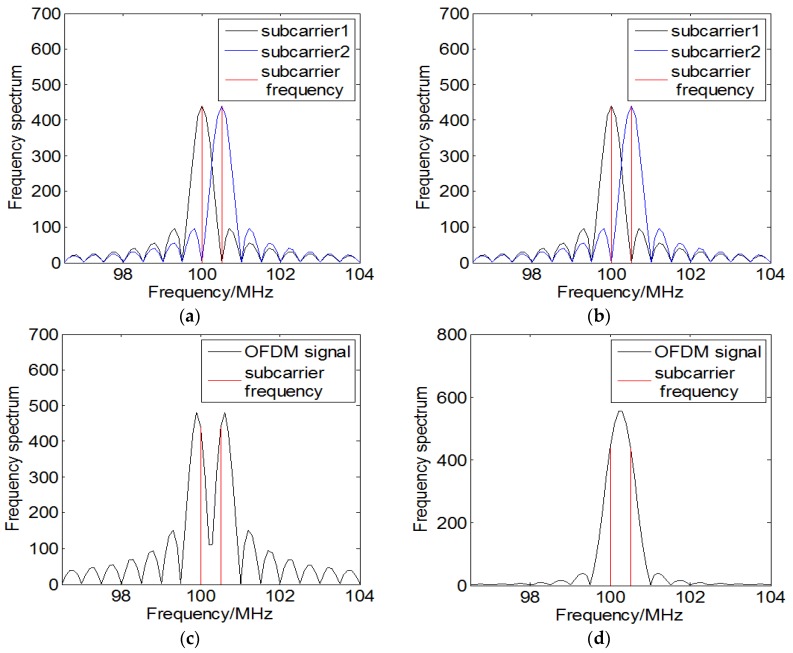

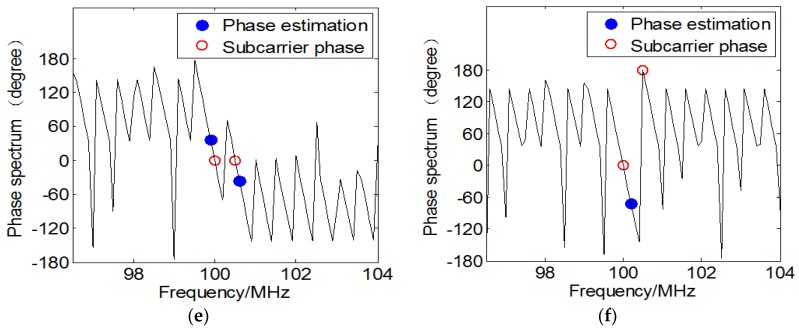
The effect of the subcarriers’ frequencies and phases on spectrum by performing FFT algorithm: (**a**) Subcarrier frequency spectrum (Δϑ=0); (**b**) Subcarrier frequency spectrum (Δϑ=π); (**c**) Received data frequency spectrum (Δϑ=0); (**d**) Received data frequency spectrum (Δϑ=π); (**e**) Received data phase spectrum (Δϑ=0); (**f**) Received data phase spectrum (Δϑ=π).

For the received data of the sensors under UCA, considering that the OFDM source is a form of mono-frequency source that needs to consider the impact of frequency, the conventional method employs the FFT algorithm to obtain the frequency spectrum and estimate the frequencies of subcarriers. Although padding zeroes at the tail of the discrete measurements is equal to interpolating in the frequency domain, which can improve frequency resolution and avoid the phenomenon of spectrum leakage, it will lead to the inaccuracy estimation of frequency as well as the corresponding phase. Take the first sensor’s received data for example, considering that an OFDM signal contains two subcarriers whose phase-code ϑ is same and frequency interval is 0.5 MHz, the subcarriers’ frequency spectrum by performing the FFT algorithm under noiseless condition is shown in Figure 3a, where the black curve and the blue curve represent the frequency spectrum of the two subcarriers respectively. It can be noticed that the subcarrier’s spectrum has half overlap with each other, which reflects the orthogonality of the OFDM signal’s subcarriers. The frequency spectrum of the OFDM signal is shown in Figure 3c, where the red lines represent the real frequencies of subcarriers. Because of the disturbance of the main and side lobes to the other subcarrier’s spectrum, the peaks of the frequency spectrum is deviated from the actual values. The phase spectrum is shown in Figure 3e, where the red hollow dots represent the real phases of subcarriers and the blue solid dots represent the corresponding phases of the frequency spectrums’ peaks. It can be seen that the phase-code of the subcarriers is equal but the estimated phases is deviated from the true values. Besides, when the difference of phase-code Δϑ is π and the frequency interval is 0.5 MHz, the frequency spectrum and phase spectrum are shown in Figure 3b,d,f. It can be noticed from Figure 3d that the subcarriers cannot be resolved from the peak in the frequency spectrum.

In order to overcome the drawbacks of the frequency spectrum by employing the FFT algorithm and obtain the super-resolution frequencies of the OFDM signal’s subcarriers for the phase-based algorithm, this paper utilizes the sparse representation to decouple subcarriers in the frequency domain. The goal of the paper is to explore how to employ the sparse representation methodology to obtain the super-resolution frequency spectrum of the OFDM signal. Considering that the received data of each sensor under UCA contains the same components of frequency, we employ the first sensor’s received data $x_{1}$ to obtain the super-resolution frequencies of the OFDM signal’s subcarriers, which can be expressed as

(6) $$x_{1}=\Phi y$$

where

(7) $$x_{1}={\left[x_{1}\left[1\right],x_{1}\left[2\right],\cdots ,x_{1}\left[N\right]\right]}^{T}$$

(8) $$y={\left[y\left[1\right],y\left[2\right],\cdots ,y\left[N_{y}\right]\right]}^{T}$$

(9) $$\Phi =\frac{1}{N_{y}}{\left[\begin{array}{cccc} 1 & 1 & \cdots & 1\\ 1 & e^{j(2\pi /N_{y})\cdot 1\cdot 1} & \cdots & e^{j(2\pi /N_{y})\cdot 1\cdot (N_{y}-1)}\\ \vdots & \vdots & \vdots & \vdots \\ 1 & e^{j(2\pi /N_{y})\cdot (N-1)\cdot 1} & \cdots & e^{j(2\pi /N_{y})\cdot (N-1)\cdot (N_{y}-1)} \end{array}\right]}_{N\times N_{y}}$$

${\left(•\right)}^{T}$ denotes the transpose operator, $\Phi \in {\mathbb{C}}^{N\times N_{y}}$ is a known matrix which is referred to as a dictionary with $N<N_{y}$, $N_{y}$ is the dimension of vector y, y refer to a representation of the received data $x_{1}$ with respect to the dictionary, which is sparse if there are few non-zeroes among the possible entries. Generically, this formulation is referred to as sparse approximation. The assumption of sparsity of y is significant when we try to obtain the unique solution because the problem in (6) is ill-posed. Only when y is sparse enough can we achieve the precise estimation of the frequencies and phases of the OFDM signal.

In order to obtain sparse representation y in (6), considering the problem in (6) is ill-posed and has many solutions, we attempt to solve an optimization problem of the following form

(10) $$\min{\left\|y\right\|}_{1}\;s.t{\left\|x_{1}-\Phi y\right\|}_{2}^{}\leq \varepsilon$$

where ‖·‖ denotes the Euclidean norm for vectors, ${\left\|\cdot \right\|}_{1}$ represents $\ell_{1}$-norm, ${\left\|\cdot \right\|}_{2}$ represents $\ell_{2}$-norm, which forces the residual $x_{1}-\Phi y$ to be smaller than a threshold ε. Therefore, y refer to the super-resolution frequency spectrum, and the frequency estimation of the pth subcarrier can be obtained as

(11) $${\hat{f}}_{p}=f_{s}k_{p}/N_{y}$$

where $f_{s}$ is the sampling frequency, $k_{p}$ is the position of the pth peak in y. By turning to sparse representation framework, we are able to achieve super-resolution in the frequency domain. Further, the estimated frequencies can be utilized to calculate the corresponding phases of the received data.

## 4. 3-D Parameter Estimation of the OFDM Signal

In order to obtain 3-D position of the OFDM signal, this paper utilizes the phase difference of the adjacent sensors in a matrix form to estimate the 3-D parameters.

The phase of the pth subcarrier at the mth sensor can be estimated as

(12) $$\begin{array}{ll} \eta_{m,p} & =\arg\left(X_{m}(k_{p})\right)\\ & =\left(2\pi {\hat{f}}_{p}R/c\right)\left\{\zeta_{m,p}(\phi_{p},\theta_{p})-\left(R/2r_{p}\right)\left(1-\zeta_{m,p}{\left(\phi_{p},\theta_{p}\right)}^{2}\right)\right\}+2\pi q \end{array}$$

for m=1,2,…,M, where $X_{m}\left(k_{p}\right)$ represents the value of the pth peak in frequency spectrum by performing sparse representation at the mth sensor, c is speed of light, q is a definite integer. The phase difference of the adjacent sensors for the pth subcarrier can be written as

(13) $$\begin{array}{ll} u_{m,p} & =\eta_{m,p}-\eta_{m+1,p}\\ & =\left(2\pi {\hat{f}}_{p}R/c\right)\left\{\left[\zeta_{m,p}(\phi_{p},\theta_{p})-\zeta_{m+1,p}(\phi_{p},\theta_{p})\right]+\left(R/2r_{p}\right)\left[\zeta_{m,p}^{2}(\phi_{p},\theta_{p})-\zeta_{m+1,p}^{2}(\phi_{p},\theta_{p})\right]\right\}+2\pi q_{1} \end{array}$$

for m=1,2,…,M−1, where $\zeta_{m}^{}(\phi ,\theta )=\cos(\gamma_{m}^{}-\phi )\sin\theta$ with $\gamma_{m}^{}=2\pi (m-1)/M$, $q_{1}$ is a definite integer. The phase ambiguity problem has already been well addressed in [9]. To guarantee no phase ambiguity in $u_{m,p}$, the condition R≤λ/4 is assumed to ensure $u_{m,p}\in [-\pi ,\pi )$. By observing (13), when $q_{1}=0$, there is no phase ambiguity about the adjacent sensors’ phase difference. When $q_{1}\neq 0$, according to the method in [9], we can employ virtual short baseline formed by the same sensor before and after rotation instead of that of the adjacent sensors. Due to the fact that the short-based-line is less than the maximal rotation angle, which can avoid phase ambiguity in the phase-based method.

It can be noticed that $\zeta_{m}^{}(\phi ,\theta )$ can be decomposed by employing angle transformation formula of trigonometric function, and $\zeta_{m,p}^{2}(\phi_{p},\theta_{p})$ by employing the double angle formula of trigonometric function. Therefore, by extending the scheme in [8] to decouple the phase difference $u_{m,p}$, we reformulate (14) as the form of matrix

(14) u=Ab

where

(15) $$u=\frac{c}{2\pi R}{\left[u_{1},u_{2},\ldots ,u_{M-1}\right]}^{T}$$

(16) $$A=\left[\begin{array}{cccc} \cos(\gamma_{1}^{})-\cos(\gamma_{2}^{}) & \sin(\gamma_{1}^{})-\sin(\gamma_{2}^{}) & \cos(2\gamma_{1}^{})-\cos(2\gamma_{2}^{}) & \sin(2\gamma_{1}^{})-\sin(2\gamma_{2}^{})\\ \cos(\gamma_{2}^{})-\cos(\gamma_{3}^{}) & \sin(\gamma_{2}^{})-\sin(\gamma_{3}^{}) & \cos(2\gamma_{2}^{})-\cos(2\gamma_{3}^{}) & \sin(2\gamma_{2}^{})-\sin(2\gamma_{3}^{})\\ \vdots & \vdots & \vdots & \vdots \\ \cos(\gamma_{M-1}^{})-\cos(\gamma_{M}^{}) & \sin(\gamma_{M-1}^{})-\sin(\gamma_{M}^{}) & \cos(2\gamma_{M-1}^{})-\cos(2\gamma_{M}^{}) & \sin(2\gamma_{M-1}^{})-\sin(2\gamma_{M}^{}) \end{array}\right]$$

(17) $$b=\left[\begin{array}{c} \cos(\phi )\sin(\theta )\\ \sin(\phi )\sin(\theta )\\ \left(R/4r\right)\cos(2\phi )\sin^{2}(\theta )\\ \left(R/4r\right)\sin(2\phi )\sin^{2}(\theta ) \end{array}\right]$$

for m=1,2,…,M−1, where $u_{m}=\frac{1}{P}\sum_{p=1}^{P}u_{{}_{m,p}}/{\hat{f}}_{p}$ represents the average of all subcarriers’ phase differences at the mth sensor. The method in [8] for the single-frequency source can be promoted and employed to estimate the individual subcarrier’s 3-D localization, which approximately represents the 3-D localization of the OFDM signal. However, the method in [8] does not make full use of subcarriers’ frequencies and phase differences. It can be noticed that the improved algorithm utilizes the normalized phase differences of the OFDM signal’s subcarriers, which can obtain precise localization of the near-field OFDM source.

Note that only the matrix b includes the parameters of azimuth angle, elevation angle and range. By using the least square method, the optimal solution of b can be estimated as

(18) $$\hat{b}={\left[\begin{array}{cccc} {\hat{b}}_{1} & {\hat{b}}_{2} & {\hat{b}}_{3} & {\hat{b}}_{4} \end{array}\right]}^{T}={\left(A^{T}A\right)}^{-1}A^{T}u$$

As a result, the estimation of the OFDM signal’s azimuth angle, elevation angle and range are respectively obtained from (16)

(19) $$\phi =\arg\left\{{\hat{b}}_{1}+j{\hat{b}}_{2}\right\}$$

(20) $$\theta =\arcsin\left\{\sqrt{{\hat{b}}_{1}{}^{2}+{\hat{b}}_{2}{}^{2}}\right\}$$

(21) $$r=\frac{R}{4}\frac{{\hat{b}}_{1}{}^{2}+{\hat{b}}_{2}{}^{2}}{\sqrt{{\hat{b}}_{3}{}^{2}+{\hat{b}}_{4}{}^{2}}}$$

**Figure 4:**
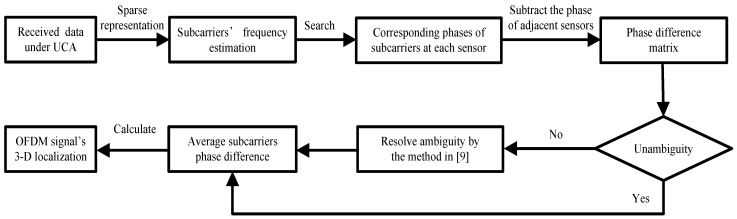
Flow chart of the proposed phase-based algorithm.

The flow chart of the proposed closed-form algorithm based on phase difference for a near-field OFDM signal’s 3-D localization is shown in Figure 4.

## 5. Simulation Results

In this section, simulations are performed to demonstrate the effectiveness of our proposed algorithm. The results show that the proposed algorithm can accurately estimate the frequencies and the corresponding phases of the OFDM signal’s subcarriers as well as the 3-D position of the near-field OFDM signal.

### 5.1. Performance of Sparse Representation

In order to verify that sparse representation can decouple the subcarriers of the OFDM signal in the frequency domain and obtain the corresponding phases, we consider a simulation that the OFDM signal has 8 subcarriers and the carrier frequency $f_{c}=100\;\mathrm{MHz}$. After the processing of the up converters, the minimal frequency of subcarrier is 100 MHz and the frequency spacing is 0.5 MHz, which belongs to the wideband source. Besides, the subcarrier’s phase in the duration of the OFDM elementary symbol is modulated by employing 8 Phase Shift Keying (8PSK), which is shown in Table 1. For all examples, we set an eight-sensor symmetric UCA with radius R=0.6 m. Due to the fact that the maximum frequency of the subcarrier is 103.5 MHz and the corresponding minimum wavelength is 2.90 m, it is can be noticed that the radius is less than the a quarter of the minimum wavelength, which can guarantee that there is no ambiguity for the phase-based algorithm. The sampling frequency and snapshot number are set at 2 GHz and 4000, respectively. At the same time, the FFT algorithm is executed to perform the comparison of the frequency estimation. 

**Figure 5:**
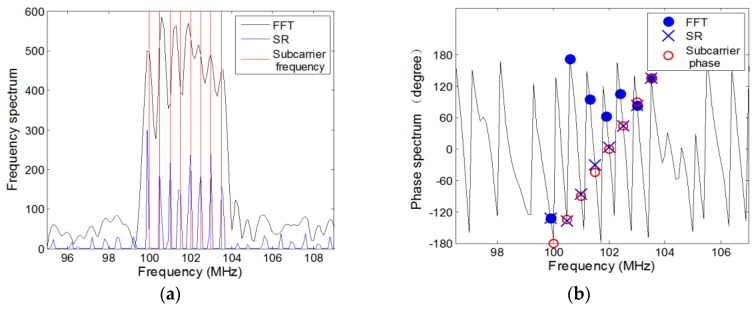

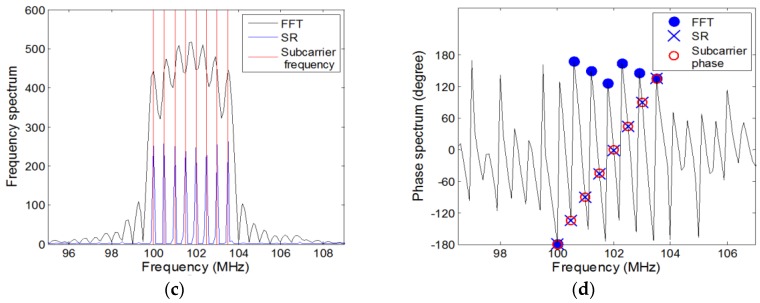
OFDM signal spectrum: (**a**) Frequency spectrum (SNR = 0 dB); (**b**) Phase spectrum (SNR = 0 dB); (**c**) Frequency spectrum (SNR = 20 dB); (**d**) Phase spectrum (SNR = 20 dB).

When the signal-to-noise ratio (SNR) is set at 0 dB and 20 dB, the frequency spectrum of the first sensor’s received data are respectively shown in Figure 5a,c, where the red lines represent the real frequency of subcarriers, the black curve represents the frequency spectrum by employing the FFT algorithm through padding zeros at the tail of the discrete received data, and the blue curve represents the frequency spectrum by performing sparse representation. It is noteworthy that the peaks by employing the FFT algorithm do not correspond to the actual values but the peaks of the sparse representation can estimate the subcarriers’ frequencies accurately. The phase spectrum are shown in Figure 5b,d, where the red hollow dots represent the real phases of subcarriers, the blue solid dots represent the corresponding phases of the peaks in the frequency spectrum by employing the FFT algorithm and the × shape dots represent the corresponding phases of the peaks by employing sparse representation. It is can be seen that the FFT algorithm loses efficacy for the frequency and the corresponding phase estimation but the results of the proposed sparse representation is corresponding to the actual values. The conclusion could be reached that the proposed method of sparse representation is superior in the performance for estimating the frequencies and the corresponding phases for the OFDM signal.

### 5.2. Performance of Location

In this section, in order to demonstrate the superior 3-D parameter estimation performance of the proposed algorithm, due to the fact that the method in [8] for the single-frequency source can be promoted and employed to estimate the individual subcarrier’s 3-D localization, which approximately represents the 3-D localization of the OFDM signal, the first and the fourth subcarriers’ localization results by employing the method in [8] are executed to perform the comparison. The results are evaluated by employing the estimated root mean square errors (RMSEs) from the average results of 500 independent Monte Carlo simulations for the mentioned OFDM signal located at $\left(20^{\circ },50^{\circ },6\;m\right)$.

**Figure 6:**
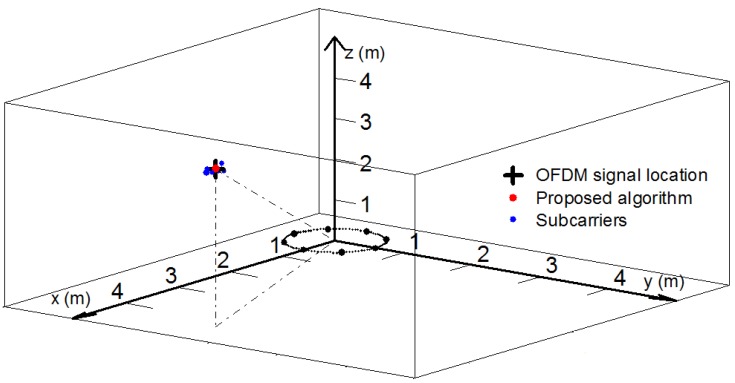
Positioning result (SNR = 20 dB).

When the SNR is set as 20 dB, the *p*th subcarrier’s wavelength $\lambda_{p}$ in [8] can be calculated by $c/f_{p}$, where c is speed of light and $f_{p}$ is the *p*th subcarrier’s frequency. The 3-D position simulation results are shown in Table 2 and Figure 6. Due to the fact that the method in [8] only considers the single-frequency source which can be referred to as the narrow-band, but the OFDM source is a form of mono-frequency source, which needs to consider the impact of frequency, it can be seen from Table 2 that the result of the proposed method employed the normalized subcarriers’ phase differences is closer to the actual location, and the performance of the proposed method is superior to the method in [8].

As shown in Figure 6, the + shape represents the OFDM signal real location, the red dot represents the location of the proposed method and the blue dots represent the locations of the individual subcarriers’ locations by employing the method in [8]. Due to the fact that the method in [8] is only suitable for the single-frequency source, it is can be noticed that the positioning results of the individual subcarriers are distributed around the OFDM signal’s real location, and the proposed algorithm with comprehensive utilization of the subcarriers’ phase differences and frequencies can achieve a more precise location.

**Figure 7:**
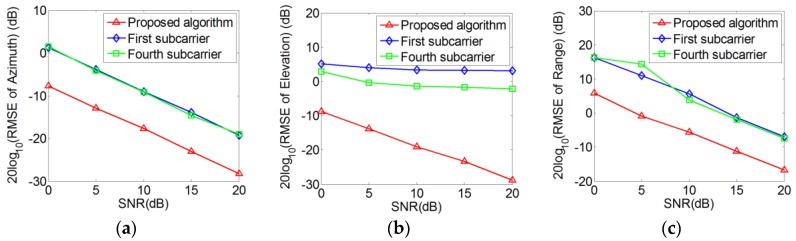
RMSEs versus SNR: (**a**) Azimuth angle; (**b**) Elevation angle; (**c**) Range.

In order to further demonstrate the superiority of the proposed method for the near-field OFDM signal localization, we compare the RMSEs of the proposed algorithm to that of the method by directly employing the individual subcarrier alone and the method in [8]. The logarithm of the RMSEs are shown in Figure 7, where the red lines represent the parameter estimation of the proposed method, the blue lines represent the fourth subcarrier’s parameter estimation by employing the method in [8] and the green lines represent the first subcarrier’s parameter estimation by employing the method in [8]. As the proposed algorithm makes full use of subcarriers’ phase differences, it can be noticed that the proposed algorithm can obtain the near-field OFDM signal’s location effectively with increased SNR. Moreover, although there are errors in the frequency estimation by employing sparse representation under low SNR, it can be seen that the improved method for the localization of the OFDM signal can reduce errors and the performance is superior to method in [8] especially for elevation angle.

## 6. Conclusions

A closed-form algorithm based on phase difference is proposed for the 3-D location (azimuth angle, elevation angle and range) of the near-field OFDM signal under UCA. The proposed algorithm synthetically utilizes the estimated subcarriers’ phase differences of the adjacent sensors and employs the least square method to acquire precise 3-D parameters of the OFDM signal’s location, which promotes the phase-based method in [8] for a single-frequency source to the specific practical application of the OFDM source. Moreover, as the mono-frequency estimation is significant to the phase-based method and the existing method cannot resolve the frequencies of the OFDM signal’s subcarriers in the frequency domain, the advantage of the proposed algorithm is that it employs sparse representation to obtain the super-resolution frequencies and the corresponding phases of the OFDM signal’s subcarriers in the case of unknown signal’s parameters. Compared to the individual subcarriers’ localization results by directly employing the method in [8], the proposed algorithm makes full use of subcarriers’ phase differences and has superior performance for the 3-D parameter estimation especially for elevation angle.

Due to the fact that the subcarriers of the OFDM source can be referred to as the single-frequency sources and the far-field source can be referred to as the condition that the near-field source’s range is infinite, the proposed algorithm can be also applicable to the localization of parameter estimation in the case of the multiple near-field mono-frequency sources or a far-field OFDM signal.

## Figures and Tables

**Table 1:** The frequencies and phases of the OFDM signal’s subcarriers.

Subcarrier	1	2	3	4	5	6	7	8
Frequency (MHz)	100	100.5	101	101.5	102	102.5	103	103.5
Phase (rad)	−π	−3π/4	−π/2	−π/4	0	π/4	π/2	3π/4

**Table 2:** 3-D parameter estimation comparison.

Parameter Estimation	Actual Parameter	Proposed Method	Subcarrier ^1^ 1	Subcarrier ^1^ 4
Azimuth angle (degree)	20	20.01	20.17	20.05
Elevation angle (degree)	50	49.98	50.78	51.51
Range (m)	6	6.01	5.57	6.80

^1^ represents the individual subcarrier’s localization by directly employing the method in [8].
